# Supplementary material for: Combined abnormal muscle activity and pain-related factors affect disability in patients with chronic low back pain: An association rule analysis
Source: PLoS One. 2020 Dec 17;15(12):e0244111. doi: 10.1371/journal.pone.0244111 (PMC7746291; doi:10.1371/journal.pone.0244111)
Supplement: Attachment — Submitted filename: Response to Reviewers.docx [file pone.0244111.s003.docx]

2-December-2020

Dr. Joerg Heber

Editor-in-Chief

*PLOS ONE*

Dear Editors:

**Manuscript ID PONE-D-20-19882, "** **Combined abnormal muscle activity and pain-related factors affect disability in patients with chronic low back pain: An association rule analysis," by Shigetoh et al.** **'**

Please find attached a revised version of our above-named manuscript, which we would like to resubmit for publication as a Research Article in *PLOS ONE*.

Your comments and those of the reviewers were highly insightful and enabled us to greatly improve the quality of our manuscript. In the following pages are our point-by-point responses to each of the comments of the reviewers as well as your own comments.

Revisions in the text are shown using red-colored font. In accord with Reviewer No.1’s suggestion, we revised text in the Methods and Discussion. We hope that the revisions in the manuscript and our accompanying responses will be sufficient to make our manuscript suitable for publication in *PLOS ONE*.

We look forward to hearing from you at your earliest convenience.

Sincerely,

Dr. Hayato Shigetoh

Department of Neurorehabilitation

Graduate School of Health Sciences, Kio University

4 Chome-2-2 Umaminaka, Koryo, Kitakatsuragi District, Nara 635-0832, Japan

Tel.: +81-745-54-1601

Fax: +81-745-54-1600

Email: [hayato.pt1121@gmail.com](mailto:hayato.pt1121@gmail.com)

**Manuscript ID PONE-D-20-19882, "** **Combined abnormal muscle activity and pain-related factors affect disability in patients with chronic low back pain: An association rule analysis," by Shigetoh et al.** **'**

**Responses to the comments of Reviewer #1**

Reviewer #1:

Thank you for addressing my comments and providing a clear outline of how this was done. I have a few additional minor comments below:

**Response: Thank you for reviewing our manuscript. Your comments were highly insightful and enabled us to greatly improve the quality of our manuscript. In the following pages are our point-by-point responses to each of the comments of the reviewers as well as your own comments. Revisions in the text are shown using red-colored font.**

Page 7, lines 113 to 115: The changes provide more detail about the study participants which will help inform readers in terms of applicability of findings to their patients, and researchers in terms of study replication or inclusion in systematic reviews. I have made some edits to these sentences and written it out as follows.

Also, the patient's LBP was categorized as non-specific LBP (n = 6) and specific LBP (n = 18). The specific LBP included spinal stenosis (n = 3) and lumbar osteoarthritis (n =15).

**Response:** We have done so. We revised the Methods section.

Page 12, line 197: please edit sentence as follows.

The patient's glabrous skin over the erector spinae was abraded and cleaned with an alcohol wipe.

**Response:** We have done so. We revised the Methods section.

Page 33, lines 490 to 498: I have made edits to these sentences, please see below.

This study had several limitations. (1) The sample size of this study is not large. A small sample size increases the probability that one patient's results will be affected by small sample size in association rule analysis (especially “support”). In the present study, the Fisher exact test was performed, one method of examining generalizability when the sample size is

small. However, the current study results did not exclude the possibility that the sample size affected the results. (2) The small sample size, the type of LBP experienced by participants, and the specific assessment techniques used limit the generalizability of this study's findings.

**Response:** We have done so. We revised the Discussion section.
